# Data driven decision making to characterize clinical personas of parents of children with cystic fibrosis: a mixed methods study

**DOI:** 10.1186/s12890-020-01202-x

**Published:** 2020-06-18

**Authors:** Rhonda D. Szczesniak, Teresa Pestian, Leo L. Duan, Dan Li, Sophia Stamper, Brycen Ferrara, Elizabeth Kramer, John P. Clancy, Daniel Grossoehme

**Affiliations:** 1 Division of Biostatistics & Epidemiology, Cincinnati Children’s Hospital Medical Center, 3333 Burnet Ave (MLC 5041), Cincinnati, OH 45229 USA; 2 Division of Pulmonary Medicine, Cincinnati Children’s Hospital Medical Center, Cincinnati, USA; 3 Department of Pediatrics, University of Cincinnati College of Medicine, Cincinnati, OH USA; 4 Department of Statistics, University of Florida, Gainesville, FL USA; 5 Alzheimer’s Therapeutic Research Institute, Keck School of Medicine, University of Southern California, Los Angeles, CA USA; 6 Division of Pediatric & Adolescent Gynecology, Cincinnati Children’s Hospital Medical Center, Cincinnati, OH USA; 7 Cystic Fibrosis Foundation, Bethesda, MD USA; 8 Haslinger Family Pediatric Palliative Care Center, Akron Children’s Hospital, Akron, OH USA; 9 Rebecca D. Considine Research Institute, Akron Children’s Hospital, Akron, OH USA

**Keywords:** Bayesian, Clustering, Cystic fibrosis, Health-care analytics, Health-care delivery, Mixed methods, Personalized medicine, Statistical learning, Theory of reasoned action

## Abstract

**Background:**

Beginning at a young age, children with cystic fibrosis (CF) embark on demanding care regimens that pose challenges to parents. We examined the extent to which clinical, demographic and psychosocial features inform patterns of adherence to pulmonary therapies and how these patterns can be used to develop clinical personas, defined as aspects of adherence barriers that are presented by parents and/or perceived by clinicians, in order to enhance personalized CF care delivery.

**Methods:**

We undertook an explanatory sequential mixed-methods study consisting of i) multivariate clustering to create clusters corresponding to parental adherence patterns (quantitative phase); ii) parental participant interviews to create clinical personas interpreted from clustering (qualitative phase). Clinical, demographic and psychosocial features were used in supervised clustering against clinical endpoints, which included adherence to airway clearance and aerosolized medications and self-efficacy score, which was used as a feature for modeling adherence. Clinical implications were developed for each persona by combing quantitative and qualitative data (integration phase).

**Results:**

The quantitative phase showed that the 87 parent participants were segmented into three distinct patterns of adherence based on use of aerosolized medication and practice of airway clearance. Patterns were primarily influenced by self-efficacy, distance to CF care center and child BMI percentile. The two key patterns that emerged for the self-efficacy model were most heavily influenced by distance to CF care center and child BMI percentile. Eight clinical personas were developed in the qualitative phase from parent and clinician participant feedback of latent components from these models. Findings from the integration phase include recommendations to overcome specific challenges with maintaining treatment regimens and increasing support from social networks.

**Conclusions:**

Adherence patterns from multivariate models and resulting parent personas with their corresponding clinical implications have utility as clinical decision support tools and capabilities for tailoring intervention study designs that promote adherence.

## Background

Cystic Fibrosis (CF) is a life-limiting autosomal recessive genetic disease characterized by poor growth and progressive obstructive lung disease. There are nearly 70,000 individuals currently living with CF worldwide [1]. With the advent of new therapeutics and quality improvement initiatives in recent decades, individuals with CF are living longer than ever before; median survival estimates from UK and US CF registries are well above 40 years of age [2, 3]. Individuals with CF who are living in developed countries are typically diagnosed within the first year of life with universal newborn screening [4]. Even at these early stages of CF, emphasis is on aggressive clinical treatment, in order to improve growth, slow lung disease progression, reduce hospitalizations and increase life expectancy.

A hallmark of CF is the burdensome daily home care management regimen; this has been attributed primarily due to the time-consuming demands of airway clearance and nebulized medication treatments [5]. A few studies have begun to shed light on adherence patterns specific to CF. Modi and colleagues applied a finite mixture model to classify treatment adherence trajectories observed over time in adolescents with CF, showing that there were low, medium and high modes of adherence to airway clearance therapies [6]. In a more recent study focused on parents of adolescents with CF based on K-means cluster analysis, we identified the existence of four modes of adherence to airway clearance therapies and three modes related to taking nebulized medications [7]. In both studies, patterns of adherence were associated post-hoc with demographic, clinical and psychosocial characteristics (known as features), indicating that religious/spiritual factors and self-efficacy are plausible contributors to adherence. Although this information may be useful for tailoring interventions to those individuals at greatest risk of poor adherence, the approaches are based on univariate, as opposed to multivariate, associations to assess differences among modes of adherence. Furthermore, clinical care outside of CF research studies do not typically include adherence tracking through Daily Phone Diaries [8] or other methods of measurement. Instead, providers rely upon clinical judgment and levels of evidence from effectiveness and efficacy studies to identify what treatments are most appropriate for a given patient.

Identifying clinical personas through advanced analytics applied to readily available encounter data with input from both caregivers and physicians has the potential to personalize care management and prioritize therapeutics development for CF care. Characterizing such clinical personas in CF has heretofore been a purely qualitative process [9]. Quantitative characterizations are often achieved through classification or cluster analysis methods. Although the two methods appear synonymous in some disciplines, the former method typically involves using a set of predefined classes and assignment of each new object to one of the classes; the latter method, which is the approach used in this study, refers to grouping a set of objects or individuals into a set of clusters based only on information found in the data, in order to describe their common characteristics and their relationships. In the statistical learning literature, these approaches are respectively termed supervised and unsupervised learning [10], and have only recently gained favor in the clinical research literature. Prominent examples are highlighted in asthma research, where disease severity is heterogeneous and clinical characteristics are complex [11, 12].

Even the state-of-art quantitative approaches have difficulty accommodating the broad types of measurements obtained in CF and other clinical populations. From a statistical standpoint, the heterogeneity arises from needing to accommodate a breadth of data types, ranging from categorical variables (e.g. gender) to continuous variables (e.g. age). To that end, we implemented specialized multivariate and mixture modeling analyses to accommodate these heterogeneous data types.

To overcome these issues, we followed goal-directed design principles, in order to develop a deeper understanding of the context in which families live when a child has CF [13]. This is a step enabling the subsequent design, prototyping, pilot testing, and implementation of pro-adherence behavioural interventions [14]. In this mixed methods study, we hypothesized that 1) quantitative development of CF clinical personas could be achieved through advanced multivariate analysis; 2) there exist distinct subgroups of CF parents/caregivers based upon observed clinical, demographic and psychosocial characteristics; 3) regimens may be tailored to these subgroups in order to promote adherence to routine CF therapies; in this context, specifically adherence to nebulized medications and airway clearance therapy. Understanding associations between clinical persons and treatment regimen adherence will allow clinicians to better tailor care regimens and provide anticipatory guidance commensurate with the individualized needs of families. Furthermore, targeting modifiable variables may allow families to better follow evidence-based treatment regimens and change the trajectory of early childhood CF lung disease.

## Methods

### Study design

We designed and conducted an explanatory sequential mixed-methods study [15, 16] consisting of three phases: i) multivariate modeling to identify patterns of adherence and self-efficacy (quantitative phase); ii) conversion of patterns into clinical personas outlining scenarios specific to parent-patient dyads (qualitative phase); iii) translation of scenarios into clinical implications/actions (integration phase).

### Quantitative phase

Retrospective data were utilized from a completed multi-site, cross-sectional study on parents of children with CF < 13 years of age at each of two pediatric CF care centers, located in the Midwestern and Southern regions of the US and accredited by the Cystic Fibrosis Foundation. Additional details on study design, enrollment criteria and measurements have been described in previous work [7]. The quantitative data collection period was from April 18, 2011, to December 4, 2013. Variable selection was theoretically grounded in the Theory of Reasoned Action [17], which posits that a behavior (in this case, adherence to prescribed therapies) is predicted by a person’s level of intention to perform the behavior. Intention is, in turn, predicted by the behavior’s perceived benefit, the behavioral norms one perceives regarding performing the behavior or not, and one’s self-efficacy to actually complete the behavior under various conditions. Those three determinants of intention are themselves predicted by a variety of “background factors” which include disease-specific factors, demographics, co-morbidities, beliefs and values. The Theory of Reasoned Action has been used to study a variety of health behaviors, and has been used to study adherence to prescribed CF therapies by parents for their children, as well as adolescent and adult adherence to their own therapies [7, 18, 19]. All available demographic, clinical and psychosocial variables from the original study were considered; these included clinical variables collected on each child participant: Body Mass Index (BMI) percentile at the most recent visit prior to study enrollment, the number of pulmonary exacerbations within the prior year (an exacerbation was defined as use of intravenous antibiotics prescribed for respiratory symptoms at-home or in the hospital) and age at enrollment. Demographic variables obtained from parents included age, gender, education level, if the parent had > 1 child with CF, the roadway distance (in miles) from their residence to the primary CF center, which has been identified as a correlate of CF lung disease [20]. Questionnaire measures included parental use of negative spiritual coping, as measured by the Brief RCOPE [21]; degree of religiosity, measured by the Duke University Religion Index (DUREL) scale [22]; depressive symptoms, as measured by the Center for Epidemiologic Studies Depression (CES-D) [23]; self-efficacy was assessed as a determinant of adherence [24]. Survey measures have been detailed in this previous study, including reliability estimates, score ranges and examples of the questions. Adherence rates were calculated using data from the Daily Phone Diary (DPD), a validated instrument to collect adherence data [25]. These data are obtained from semi-structured phone interviews via cued recalls of the participant’s events in 5-min increments over the last 24 h. Each participant was scheduled to complete three DPDs; the number of treatments reportedly completed was averaged across the diaries. Prescribing patterns were obtained from chart review. The participant-specific adherence rate for each treatment of interest, aerosolized medications, and airway clearance, was calculated as the ratio of treatments completed per the DPD to treatment prescribed at the clinic appointment at which enrollment occurred. It was possible for both parents to participate in the study, as each parent could have his or her independent reporting of the child’s adherence and individual demographic, clinical and psychosocial characteristics. If both parents enrolled, male parents’ data were selected for this study, in order to obtain additional representation of fathers in the study cohort for facilitating persona development.

Continuous variables were each summarized as median (IQR); n (%) was used to summarize categorical measures. All analyses were implemented in *R. prior* to any multivariate analysis, multiple imputations were performed for data with missing values using the ‘mi’ package [26]. We performed principal component analyses of the explanatory variables according to groupings of the variables using a multiple factor analysis (MFA) technique for mixed (i.e., categorical and continuous) variables available from the ‘PCAmixdata’ package [27]. The variables were grouped as “child”, “parent religious/spiritual and depression” and “parent demographics.” A principal component analysis was implemented for each grouping of the variables using generalized singular value decomposition. Component maps of factor scores and loadings were used to examine relationships of variables between and within groupings. To assess consistency across results, a separate mixture model analysis was performed using a Dirichlet process prior and specification of mixed data distributions [28].

Partial least squares regression (PLSR) was used to estimate latent components corresponding to the adherence and self-efficacy outcomes and their potential predictors. PLSR is especially useful when relatively few observations are available compared to the number of potential predictors, and it is of interest to characterize the latent structure between the response and predictor variables. Four different PLSR models were fitted based on outcomes: a) both adherence outcomes (aerosolized medication and airway clearance) were jointly modeled; b) aerosolized medication adherence alone; c) airway clearance adherence alone; d) self-efficacy alone. In models (a)-(c), self-efficacy was included among the other predictors. The estimation was performed using the ‘pls’ package [29]. To determine the number of components in each PLSR model, ten-fold cross validation was used.

### Qualitative phase

Latent structures obtained from PLSR model in (a), which included both adherence outcomes, were used to develop clinical personas as follows. The number of personas to be created was set to be twice the number of principal components identified. This allowed the assignment of dichotomous values for each of the principal components to a persona, for example, living “near” or “far” from a CF Center. Based on those characteristics, candidate participants were identified from the data files. The DPD records for one or more randomly chosen participants matching each developing persona were used to synthesize a typical daily routine. Personal characteristics that were not significant components in the model (e.g., parental gender, age, race, child’s age at diagnosis), and hence were unrelated to actual parental adherence, were assigned to each persona such that they mirrored the demographics at the two participating centers. These characteristics were then used to create an empirically-grounded “story” for each persona, reflecting the child’s clinical condition, parental behaviors and emotional health, to stipulate their goals for their child. These draft personas were then given to a subset of the parents of children with CF who participated in the quantitative phase for their feedback on the extent to which the persona reflected their concerns when they were at that stage [30]. This process is known as “member checking” and enhances data validity.

### Integration phase

Persona-specific scenarios were drafted based on parental input aimed at identifying and elaborating on each persona’s demographics, clinical and psychosocial characteristics and synthesized routine. Once consensus was reached by parent participants, draft persona development and scenarios were considered complete. Clinical implications and actions were formed corresponding to each clinical persona’s scenarios through interviews with two pediatric CF clinicians. Results are reported in accordance with newly developed guidelines for mixed methods research [31]. A checklist for the study is provided (see Table S1 of supplemental material).

## Results

### Study cohort characteristics (quantitative phase)

There were 87 parent participants (Table 1), of which 31 (35.6%) arose from dyads in which both parents participated. Parents were mostly over 30 years old, female, and had attended college. Few parents had more than one child with CF and tended to reside far away from the child’s primary CF care center. Median CES-D exceeded the threshold score of 16.0, which is the commonly-accepted cut-off value for clinically significant symptoms of depression [23] and participants typically used negative spiritual coping, a particular style of spiritual coping that reflects feelings of religious disconnection, abandonment, or struggles with God. Self-efficacy and adherence scores were high, on average. Participants’ children were typically of pre-school age and had median BMI that met the Cystic Fibrosis Foundation goal of being at or above the 50th percentile [4]. Slightly more than half of the children did not have any pulmonary exacerbations reported within the year prior to enrollment. There was one participant who did not report information on adherence to airway clearance therapy, while 23% of participants did not report adherence to aerosolized medications because their children were not prescribed this treatment. DPD completion ranged from 1 to 3 per patient.

**Table 1:** Characteristics of participants and their children with cystic fibrosis (quantitative phase)

**Parents (*N* = 88)**	
**Age range in years, n (%)**	
**18–25**	8 (9.2%)
**26–30**	17 (19.5%)
**31–35**	31 (35.6%)
**36–40**	18 (20.7%)
**41–45**	9 (10.3)
**46 and older**	4 (4.6%)
**Female, n (%)**	75 (86.2%)
**Education**^**c**^**, n (%)**	
**Some high school**	4 (4.7%)
**Graduated high school**	16 (18.6%)
**Some college**	29 (33.7%)
**Graduated college**	30 (34.9%)
**Graduate school**	7 (8.1%)
**Multiple children with CF, n (%)**	17 (19.5%)
**Distance to CF Center in miles, med (IQR), n**	43.0 (19.3–90.7), 87
**CES-D, med (IQR), n**	17 (9–26), 81
**DUREL, med (IQR), n**	10.5 (7–14), 86
**Negative Spiritual Coping, med (IQR), n**	1 (0–3.5), 84
**Self-efficacy, median (IQR), n**	1071 (989.5–1097.3), 84
**Adherence**^**a**^	
**Aerosolized Medications, median (IQR), n**	0.8 (0.5–1.1), 68
**Airway Clearance, median (IQR), n**	0.8 (0.5–1), 87
**Children with CF**^**b**^	
**Age in years, med (IQR), n**	4.9 (2.4–9.6), 88
**BMI percentile, median (IQR), n**	58 (35–81), 87
**Exacerbations in prior year, n (%)**	
**0**	46 (52.3%)
**1**	20 (22.7%)
**2 or more**	22 (25%)

*Abbreviations*: *BMI* Body Mass Index, *CES-D* Center for Epidemiologic Studies Depression Scale, *DUREL* Duke University Religion Index

^a^Not all subjects were prescribed aerosolized medications or airway clearance therapy during study; ^b^represents number of unique children with CF to account for instances in which both parents participated in the study; ^c^used as a proxy for socioeconomic status

#### Exploratory analyses

Bivariate analyses (Table 2) indicated that parents with multiple children with CF tended to have lower self-efficacy scores. Parents who exhibited negative spiritual coping also had poorer adherence to aerosolized medication regimens and lower self-efficacy scores. Having older children with CF was associated with poorer adherence to airway clearance and lack of self-efficacy. Children with higher BMI percentiles tended to have increased adherence to aerosolized medication regimens and improved self-efficacy scores. Principal components analysis of the explanatory variables showed that a four-component solution was optimal (Chi-square statistic: 14.4 on 11 degrees of freedom, P = 0.21). Eigen values corresponding to these components were each above 1.0. Self-efficacy and distance traveled to the CF center had high values for the PCA uniqueness index (> 0.9), followed by having multiple children with CF (0.8) and use of negative spiritual coping (0.7). Clustering individual subjects via Dirichlet process mixture modeling corroborated that there were four components present among the explanatory variables. Based on these findings, we anticipated a maximum of four components in the subsequent conditional models from the PLSR.

**Table 2:** Correlations between exposure and outcome variables (quantitative phase)^a^

	**Adherence Outcomes**		
	**Aerosolized meds**	**Airway clearance**	**Self-Efficacy**
**Parents**			
**Age**	−0.14	−0.17	−0.12
**Gender**	0.02	0.06	0.09
**Education**	−0.08	0.15	0.07
**Multiple children with CF**	−0.03	−0.01	− 0.27
**Distance to CF Center (miles)**	−0.01	0.12	−0.18
**CES-D**	−0.09	−0.13	− 0.19
**DUREL**	0.06	−0.08	0.09
**Negative spiritual coping**	−0.29	−0.07	− 0.26
**Children with CF**^**b**^			
**Age (years)**	0.01	−0.20	−0.22
**BMI percentile**	0.26	0.06	0.23
**No. of exacerbations in prior year**	0.06	0.14	0.09

*Abbreviations*: *BMI* Body Mass Index, *CES-D* Center for Epidemiologic Studies Depression Scale, *DUREL* Duke University Religion Index

^a^Not all subjects were prescribed aerosolized medications or airway clearance therapy during study. Correlations are based on individuals prescribed both aerosolized medication and airway clearance. The correlations involving two continuous variables are reported as Pearson’s r, while those involving a categorical variable are expressed as polychoric correlation coefficients. ^b^Represents number of unique children with CF to account for instances in which both parents participated in the study

#### Segmentation models

Joint PLSR of both adherence outcomes (airway clearance and self-efficacy) indicated that a three-component solution explained about 98.5% of the variation between adherence outcomes and the parent/child predictors (Table 3, adherence outcome, Model 1 results). These three components corresponded to parental capability (component I) and barriers to CF care/child nutrition (components II/III). The three components indicated that self-efficacy, distance travelled to the CF center, and child BMI percentile were unique and strong predictors of overall adherence. Self-efficacy was a key driver in the first component (parental capability) and explained the most variation, while miles to CF center was most influential in the second and third components (labelled barriers to care and child nutrition, respectively); BMI percentile negatively loaded on the second and third components.

**Table 3:** Latent factors and clinical relevance from PLSR models of adherence and self-efficacy (quantitative phase)^a^

	**Adherence (Model 1)**			**Self-Efficacy (Model 2)**	
**Components (clinical relevance)**	**I** **(Parental capability)**	**II / III** **(Barriers to CF care / child nutrition)**		**I / III** **(Barriers to CF care / child nutrition)**	
**Cumulative % of variation explained by each component**	57.0%	81.0%	98.5%	68.2%	96.3%
**Characteristics**					
**Child BMI percentile**		−0.43	−0.47	0.33	0.61
**Distance to CF center (miles)**	0.12	1.24	−0.88	−1.26	0.83
**Self-efficacy**	1.03	−0.29		–	–

*Abbreviations*: *BMI* Body Mass Index, *CF* Cystic Fibrosis, *PLSR* Partial Least Squares

^a^All explanatory variables were included as appropriate but are not reported here due to small magnitudes of the loadings. Adherence model includes aerosolized medication and airway clearance adherence as outcomes

**Fig. 1:**
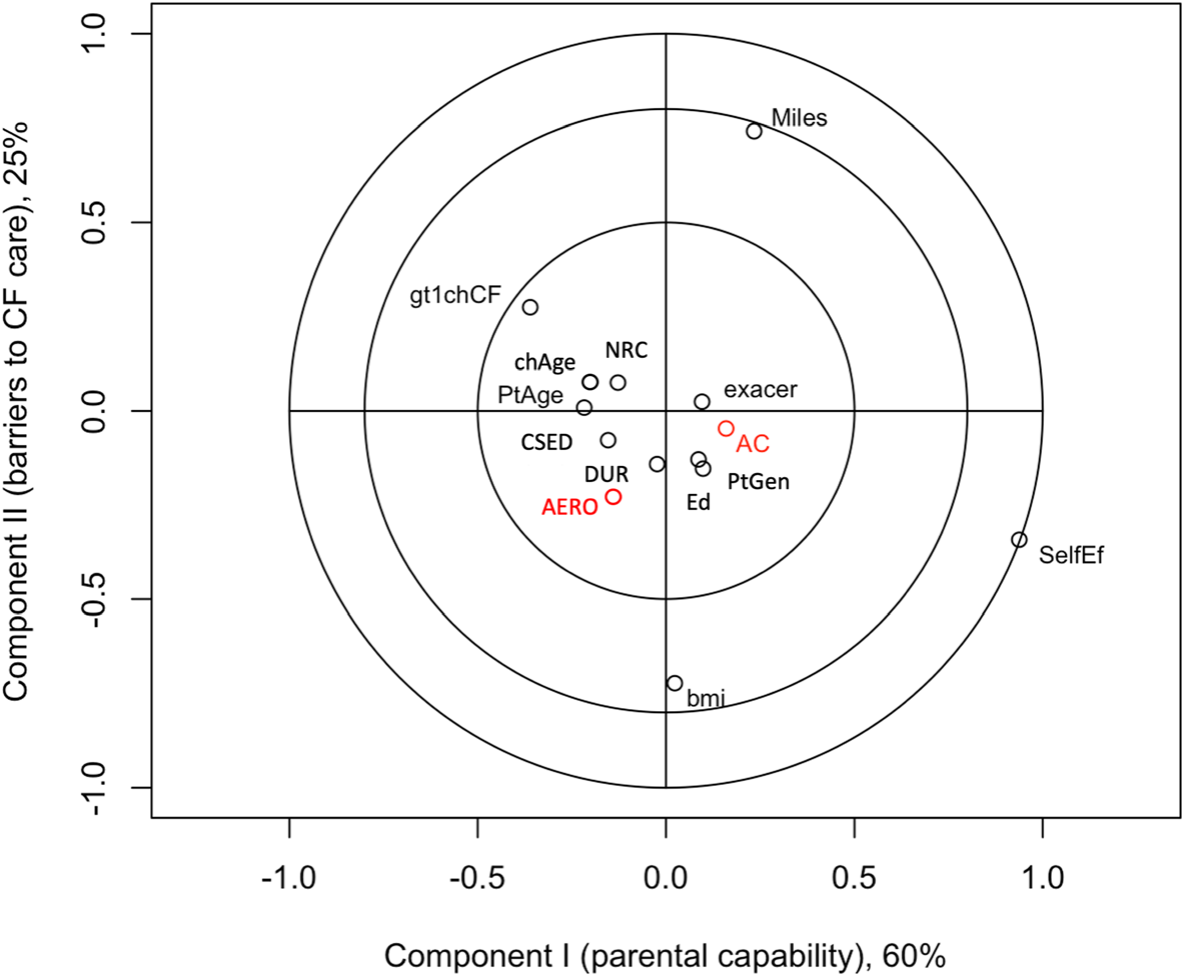
Combined adherence versus top latent clinical components from PLSR model. Corresponds to a partial least squares regression of combined adherence (aerosolized medication and airway clearance). Outcomes are labelled in red text as AERO and AC, respectively. Input variables labelled in black text are abbreviated as body mass index and age of child (bmi and chAge, respectively); number of child’s pulmonary exacerbations in prior year (exacer); parent having more than one child with CF (gt1chCF); distance travelled to CF center (Miles); self-efficacy (SelfEff); gender, age and education level of parent (PtGen, PtAge and Ed, respectively); degree of religiosity (DUR); extent of negative spiritual coping (NRC); parent depression score (CESD). Inputs contributing unique explanatory value to these two outcomes are located on outermost circles, suggesting that self-efficacy is the primary predictor of combined adherence, followed by distance travelled to CF center and body mass index

**Fig. 2:**
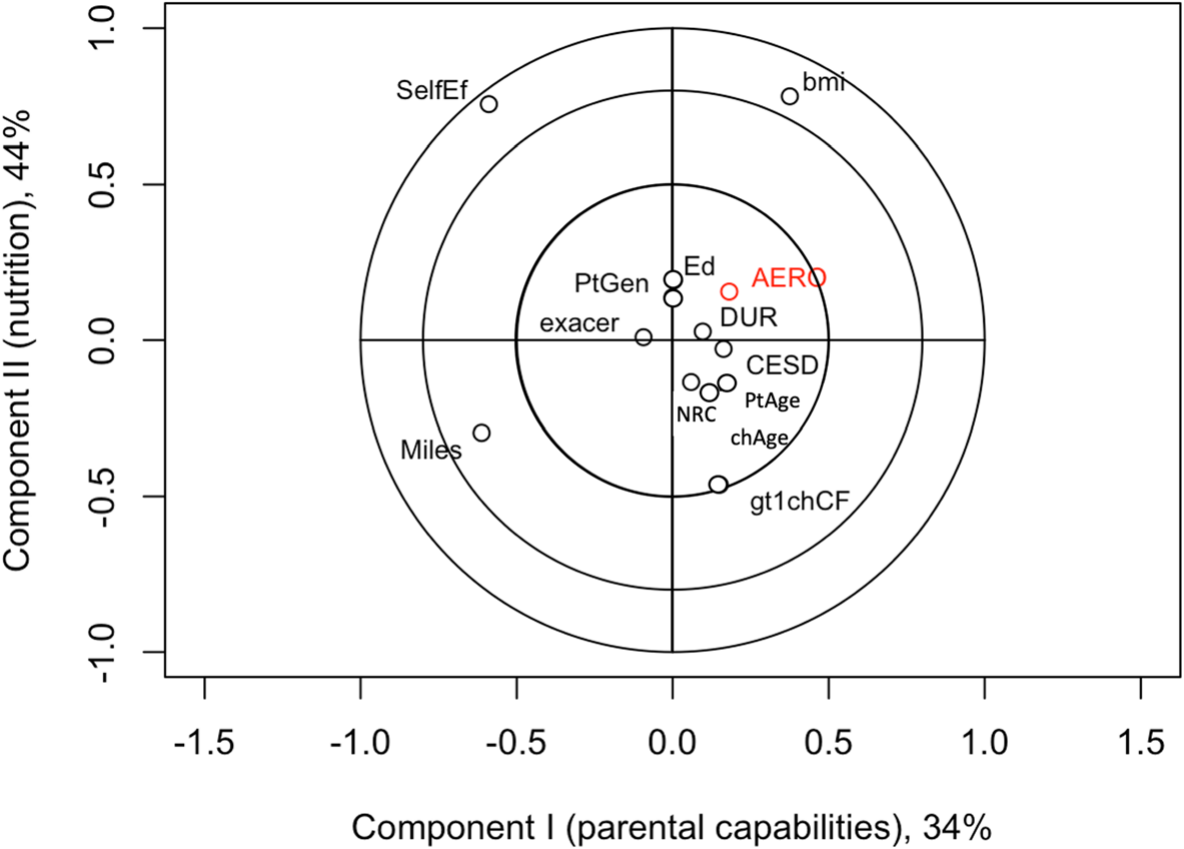
Adherence to aerosolized medication versus top latent clinical components from. PLSR model. Corresponds to a partial least squares regression of adherence to aerosolized medication only (outcome labelled as AERO). Input variables (black text) are abbreviated as in Fig. 1. Inputs contributing unique explanatory value to an outcome are located on outermost circles, suggesting that parent self-efficacy and child body mass index are the primary predictors of adherence to aerosolized medication

**Fig. 3:**
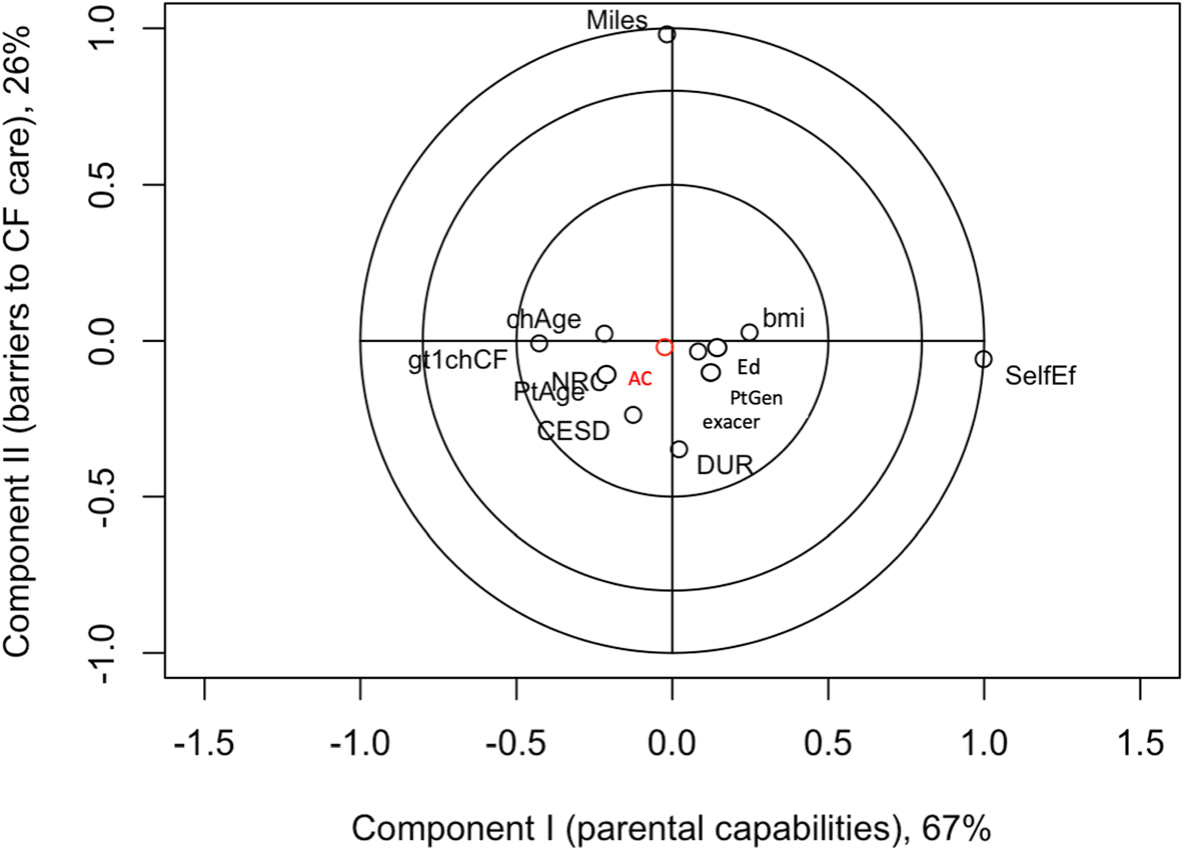
Adherence to airway clearance regimen versus top latent clinical components from PLSR model. Corresponds to a partial least squares regression of adherence to airway clearance only (outcome labelled as AC). Input variables (black text) are abbreviated as in Fig. 1. Inputs contributing unique explanatory value to an outcome are located on outermost circles, suggesting that parent self-efficacy and distance travelled to the CF center for care are the primary predictors of adherence to aerosolized medication

**Fig. 4:**
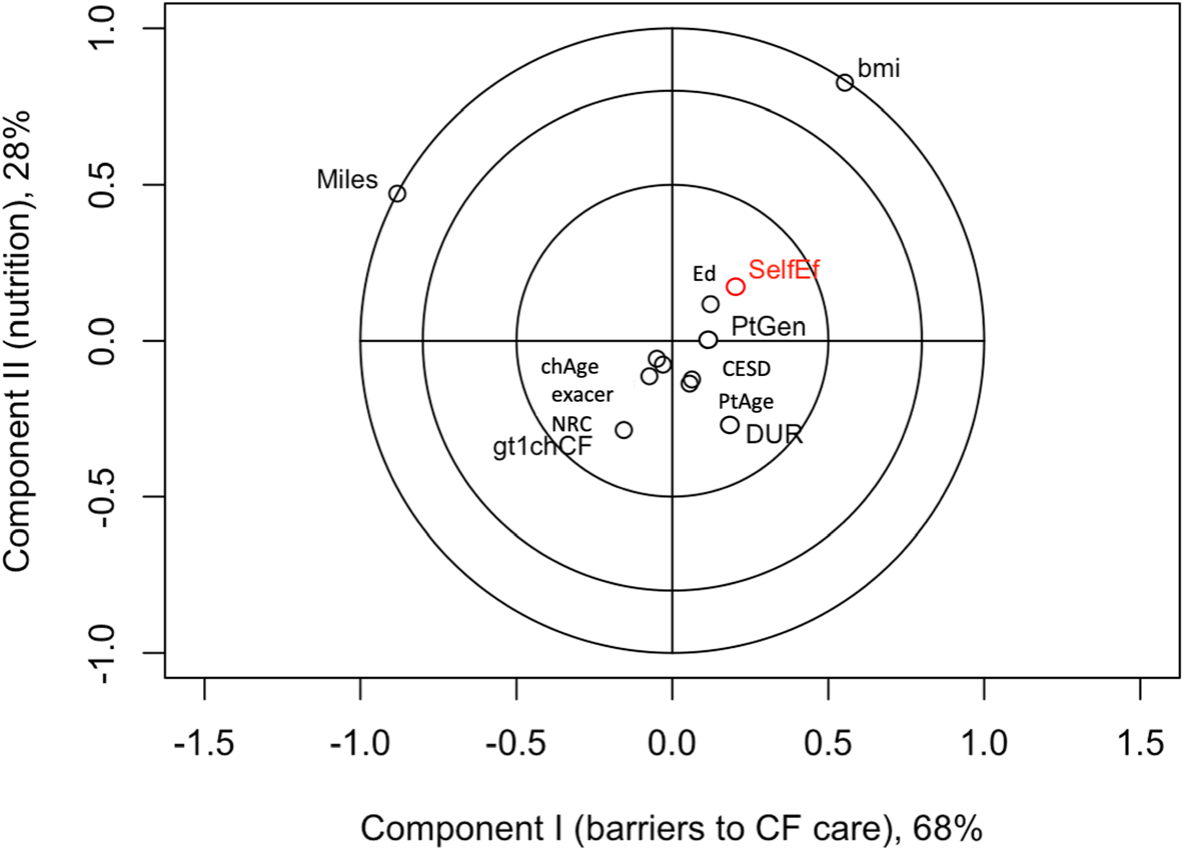
Self-efficacy versus top latent clinical components from PLSR model. Corresponds to a partial least squares regression of the outcome, degree of self-efficacy (outcome labelled as SelfEf). Input variables (black text) are abbreviated as in Fig. 1, but SelfEf appears as red text, since it is the outcome in this model. Inputs contributing unique explanatory value to an outcome are located on outermost circles, suggesting that child body mass index and distance travelled to receive care at the CF center are the primary predictors of adherence to aerosolized medication

The correlation circles of the adherence and self-efficacy outcomes and parent/child characteristics are based on association with factor scores from the first two components under PLSR Models (Figs. 1, 2, 3 and 4). Results for the joint adherence model in Fig. 1, which have each adherence outcome in red text, confirm the most influential variables reported in Table 3 and suggest that remaining clinical, demographic and psychosocial characteristics didn’t contribute unique information to the model.

Lone PLSR modeling of the adherence outcomes had similar conclusions to the joint PLSR but there were additional nuanced variables found to have some importance (Figs. 2 and 3). A three-component PLSR model explained 98.9% of the variation in the relationship between adherence to aerosolized medications and the predictor variables. Among these components, the coefficients with the largest magnitudes in the PLSR were child BMI percentile, miles traveled to CF center, and self-efficacy (Fig. 2). This separate model’s correlations between the predictor variables and scores were consistent with the joint PLSR model. The results for the PLSR modeling of adherence to airway clearance had slight differences from the joint model. Loadings indicated that the first component, which explained 67.1% of the variation between the outcome and predictors, was dominated by self-efficacy; the second component, responsible for 26.3% of variation, was comprised of miles to CF center and less intense weighting with self-efficacy; finally, the third component, although only explaining 3.2% of variation, was multifaceted, including a positive loading with child BMI percentile and smaller negative loadings with DUREL score, negative spiritual coping, CESD score and miles to CF center (Fig. 3).

The model of self-efficacy against the remaining explanatory variables indicated that there were two key components (Table 3, self-efficacy outcome, Model 2 results). These components were labelled as parental capability (component I) and barriers to CF care / child nutrition (components II/III) and collectively explained 96.3% of the variation between self-efficacy and the explanatory variables. Child BMI percentile positively loaded on both the first and second components; miles to the CF center negatively loaded on the first component and positively loaded on the second component. Correlations also reflect the dominance of these two variables (Fig. 4).

#### Clinical personas (qualitative phase)

Eight unique parent personas were constructed based on the four latent classes that emerged from the PLSR findings according to higher and lower degrees of expression for each measured variable (Table 4). There were four parent participants who provided feedback on the emerging components in a focus group setting. Participant feedback consisted of a step is known as “member-checking.” In qualitative methodology, member-checking is utilized to establish credibility of the research. Numbers of this participant size in qualitative member-checking are typical [32]. Parent personas ranged in age but tended to be older (26–38 years old). Their children with CF ranged from infants to young adolescents. The scenarios focused on complexity of routines resulting from longer distance from residence to the CF center and coordination of treatment regimen with a co-parent or the need to facilitate treatments as a single parent. Detailed characteristics of the eight parent personas are provided in Table 5 (first column).

**Table 4:**
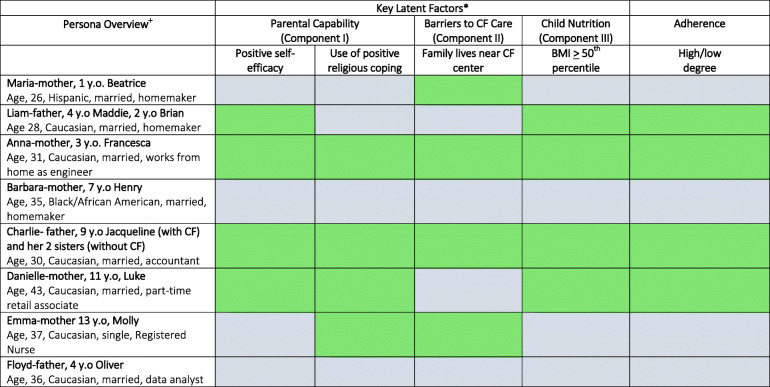
Emergent clinical personas (fusion of quantitative and qualitative phases)

*Components obtained from partial least squares regression models in the quantitative phase (Table 3). ^**+**^Personas established during fusion of qualitative/quantitative phases. Detailed persona descriptions provided in Table 5. Cells shaded reflect higher (solid light green) or lower (light blue mesh) levels of primary markers used to establish components: self-efficacy is positive/negative and parent practices positive/negative religious coping (Component I), distance traveled to CF center is high/low (Component II), child body mass index is above or below the 50^th^ percentile (Component III)

**Table 5:** Descriptions and implications of clinical personas (integration phase)

**Persona**	**Goals**	**Characteristics**	**Clinical Implications/Actions** **(Integration Phase)**
**Maria-mother, 1 y.o. Beatrice** Age, 26, Hispanic, married, homemaker	• Child will become more independent • Child is happy and has a family of her own • Illustrative quote “I don’t put her in a bubble, what’s the point? We don’t limit her in what we let her do because of CF.”	• Minimal community support through social media and church • Live 6 miles from CF center • Regularly forgets enzymes and treatments • Child’s BMI in 30th percentile • Eats breakfast with family every AM • Often naps mid-day	• Immediate focus needed on familial support. • Medical intervention is not as important as mental health • Because family lives near the CF center, clinicians could offer monthly visits and coordinate with social workers and staff psychologists if applicable
**Liam-father, 4 y.o Maddie, 2 y.o Brian** Age 28, Caucasian, married, homemaker	• Children should be true to themselves, make good decisions, and live to be an old age • Illustrative quote “We just treat them like every day is their last.”	• Active on social media for community support, but not in church • Live far from CF Center • Anxious about getting in every treatment, highly adherent • Children’s BMI in 60th Percentile • Regularly plays outside with children • Spends time with wife each night	• Family has good adherence with CF, but concerning that they live everyday as if it is their last, which may present as anxiety • Clinicians should partner with family to make care planning a team aspect, which can help normalize CF • Offering family clinical research opportunities to enroll, in order to help alleviate some anxiety and provide sense of purpose • Ensure family is properly connected with online groups. Imperative that the family is getting proper social media information and support • Connect family with a learning network monitored by the CF center to enable them to receive proper support
**Anna-mother, 3 y.o. Francesca** Age, 31, Caucasian, married, works from home as engineer	• For Francesca to enjoy her life, get the best education, and be an outstanding adult • Illustrative quote: “CF fits in like anything else. I’m a germophobe, but I’m not OCD. That’s where my faith comes in. That’s a huge thing.”	• Not active on social media, but active in church community • Live 15 miles from CF center • Highly adherent to treatments • Child’s BMI in 50th percentile • Views relationship with husband as a team	• Family is adherent and likely to incorporate changes if needed • Type of family that should continue to receive support, but there is not a strong need to intervene
**Barbara-mother, 7 y.o Henry** Age, 35, Black/African American, married, homemaker	• Happiness • Good job later in life • Independence with treatment • Illustrative quote: “I just want him to be happy. Hopefully he’ll find a really good job and a husband or wife, whichever way he goes, and to have kids and be a good person.”	• Active in church community but not social media • Live 30 miles from CF center • Not consistently adherent with treatments • Does not plan ahead or have a regular routine • Conflicted marriage • Personal health issues, depression	• Seems family is in chaos on how to work in day to day treatments • Family tends to make spur-of-the-moment decisions, a likely reason they are forgetting to be adherent • Important that the team focus on the most important medical outcome of the child as to not overwhelm this family • Helpful to focus on improving family adherence to critical treatments, such as pancreatic enzymes • Educating this family is important; since they live further away from the CF center, offer telehealth with a dietitian and social worker to provide needed support • Inform family on financial aid options for CF care to help alleviate stressors and promote focus on care
**Charlie- father, 9 y.o Jacqueline (with CF) and her 2 sisters (without CF)** Age, 30, Caucasian, married, accountant	• That child understands the importance of eating and nutrition • Illustrative quote: “Since Jacqueline is older now, it is easier because she is independent enough to do treatments herself.”	• Active in church community, regular attends bible study • Live near CF center • Adherent to treatments, child is independent enough to do majority of treatments • Childs BMI above 50th percentile • Plans family time around CF treatments	• High functioning family does good job of adhering to medication • Daughter is becoming more independent, so team can focus on educating the child to do her own treatments • Fostering her independence can make for a smooth transition into adolescence and alleviate some stressors this family may face
**Danielle-mother, 11 y.o, Luke** Age, 43, Caucasian, married, part-time retail associate	• Typical life for child • Ability for child to have many friends • Wants Luke to be part of a sports team • Major goal is for Luke to graduate high school • Illustrative quote: “Things are just so difficult right now trying to change our lives around CF, but we are trying to make it work.”	• Not active in church or social media community • Lives 55 miles from CF center • Adherent and anxious about treatments due to Luke’s late diagnosis • Child’s BMI below 40th percentile • Feels late diagnosis makes it difficult for Luke to be involved in sports and other activities • Spend a lot of time together watching TV and making dinner as a family	• Often, when people are worried about disease progression, clinicians can see high adherence • Family still struggling with their son’s BMI. Using goal setting with providers and the child to incorporate him and the family onto the team could be helpful • Goal setting will also encourage independence with the child with appropriate oversight from the team • Family should identify long-term personal goals for Luke and work with the team to give him the medical support he needs to achieve these goals. • Offer family opportunities to discuss their situation with other families of children diagnosed later in life; may also be beneficial in order to help them adjust to this new aspect of their lives.
**Emma-mother 13 y.o, Molly** Age, 37, Caucasian, single, Registered Nurse	• Understand the importance of treatments • Begin to get into a routine • Illustrative quote: “Well, I just had to change my life a lot (since CF). The feasibility of keeping drugs on you seems to get harder and harder at school. I think we have control over it. She doesn’t really think that far ahead, she’s more day by day. CF is not a bad thing in our life, just part of our life. It’s a routine”	• Not active on social media or in church community • Live near CF center • Adherent about 50–75% of the time • Child’s BMI in 10th percentile • Feels like they are constantly busy • Difficultly getting proper nutrition, often stopping to eat fast food	• Seems family has a good handle on their CF diagnosis and views CF care as part of their life • Room for nutrition improvements, and they are struggling with completing airway clearance treatments • Given family’s busy schedule, the team should focus on helping the family reschedule their routine and restructure their day to put more emphasis on improved nutrition and fitting in treatments
**Floyd-father, 4 y.o Oliver** Age, 36, Caucasian, married, data analyst	• Oliver to have a typical life and good education • Continue to work to provide for his family • Illustrative quote: “What I’m focused on now is getting to work so I can continue to have insurance to afford healthcare.”	• Not involved in social media or church community • Live far from CF center • Mostly adherent to treatments, but not sure • Child’s BMI in 35th percentile • Works overtime most weeks • Tries to spend as much time with his son as possible • Doesn’t see lack of adherence as a problem	• Parents may come into the CF clinic and seem like they have high self-efficacy and are adherent; however, upon further probing it can be revealed that there are issues with completing treatments, as is the case with this family • Sometimes parents do not always think their lack of adherence is a problem because there is no visible evidence to support this • Offer a CT/MRI for evidence-based perception, which can show actual problems with non-adherence • Closer follow-up with this family is also recommended via monthly appointments and/or telehealth

#### Clinical implications/actions (integration phase)

Although the clinician feedback was consistent on the implications/actions corresponding to each scenario, the breadth and depth of suggested intervention varied across families (Table 5, second column). Actions included increasing clinical visits, administering psychosocial assessments, discerning areas wherein improvements are feasible (e.g., dietary changes). More detailed explanation of the personas is provided as supplemental material (Table S2).

## Discussion

The purpose of this study was to develop personas of parents of children with cystic fibrosis using i) multivariate analyses followed by ii) qualitative analyses based on parental input and iii) translation of findings into implications and recommended clinical actions. With these empirically-developed personas and clinical actions, the authors were able to acquire distinct subgroups based on demographic, clinical and psychosocial data and associate each persona with adherence to CF therapeutic interventions. To our knowledge, this is the first clinical research study to identify CF clinical personas through a rigorous multi-stage approach resulting in direct guidance for care delivery.

Other statistical procedures, related either to mixture modeling or other methods of clustering, are available and have been used in asthma research [33]. Although cluster analysis often provides new insights into clinical areas in which it is applied, all too often assumptions to utilize such analysis approaches are unmet. Non-normality is a pervasive issue in cluster analysis implementation and its presence can produce misleading results, particularly in studies with small sample sizes [34]. The approach used in the current work extends what has been applied in other clinical research areas by incorporating flexibility in data types.

This study combines both personalized and precision medicine to allow for person-centered care instead of patient-centered care. Recently, adult medicine has begun the approach to person-focused care, which focuses on the whole person including their lifestyle, environment, and family dynamics. In pediatrics, it is imperative to not only focus on the person, but the family as well since parents are usually the primary caregivers. Using goal-directed design, our findings enable intervention design teams to tailor intervention more specifically. Personas allow designers to ask how the persona would interact with the intervention, and what would make it more acceptable or more feasible, for that persona. The personas also may suggest spontaneous interventions (non-manualized) in a clinical setting by helping clinicians recognize that people are adherent and non-adherent for different reasons and require a more fine-grained approach rather than one-size-fits-all interventions. For example if a clinician recognizes a parent resembling “Maria” their focus might shift away from talking about her adherence to Beatrice’s care towards how to address Maria’s emotional well-being.

Our study focused on parents of pre-teenage children with CF, but our approach may be useful for studying parents of teenage children with CF. Independent decision-making skills on adherence and other facets emerge as CF teens transition from pediatric to adult care [35]. These parents may exhibit increased symptoms of depression, reduced self-efficacy and negative religious coping that, coupled with developmental changes in their CF teens, correspond to decreased adherence to nebulized medications and airway clearance regimens. Future studies assessing adherence from both the parent and child perspectives in this sub-population may complement efforts in CF care transition research.

Often, parents will rely on social networking for community support in CF [36]. Offering parents access to a hospital learning network allows them to foster their need for community while allowing for proper oversight of information from the CF center. Learning networks provide parents access to information regarding possible clinical research opportunities. Parents who are highly adherent in the moment should not be forgotten. Different life stages can add new stressors into the life of parents and children, especially into adolescence. Discussing and encouraging independence of the child throughout periods of low stress can prophylactically help periods of high stress.

Despite the clinical insights that were gained from the study, the expansions to statistical models and improved clustering accuracy, there are limitations to the current work. First, this mixed-methods study included a relatively small sample size for the quantitative stage. This was due to the availability of participants and the difficulty with feasibility of the daily phone diaries. Specific findings may not be generalizable to other modalities of adherence aside from the two studied (aerosolized medication and airway clearance therapy). In addition, not all correlates of adherence to either modality were captured, such as other socioeconomic status proxies aside from parent education or other correlates of CF health (e.g., genotype). Future studies may include methodologic research to combat missing data and optimize timing of electronic adherence monitoring. This may prove useful given the variability of adherence patterns even to modulator therapies that target the underlying defect of CF [37]. Another limitation is the generalizability of clusters of this study cohort to the CF clinical population. Personas are not intended for parents of patients with other life-limiting illnesses. Lastly, the present study did not account for family dyad; there were 29 parent dyads. Future study approaches could be extended to utilize hierarchical clustering [38]. Future studies should also focus on how modulators can impact adherence. With the newer implementation of modulators, further research can be done to understand how researchers can define personas given modulators.

This study is not meant to address causation of traits on adherence patterns. Instead, this provides a useful framework for clinicians to use when individualizing treatment plans and working cooperatively with families to optimize their child’s health. Clinicians can use this framework to predict which families may need more attention in regard to their personalized treatment. For example, “hyper-vigilant” parents (those with high self-efficacy scores) may have children with CF who have more frequent pulmonary exacerbations (Table 2). A similar finding has been shown with “sicker” patients being more likely to receive treatment with tobramycin but tend to have worse outcome [39]. With already limited time, understanding possible personas will allow clinicians to quickly identify which families and caregivers may be having a difficult time with adherence. We have identified certain parental traits associated with barriers to care. Having multiple children with CF is associated with lower self-efficacy, while caring for an older child with CF is associated with both lower self-efficacy and decrease adherence to prescribed airway clearance regimens. While these factors are not modifiable, this identifies a need for better partnering with these families to individualize treatment plans, recognizing the unique stressors they are under. If a clinician is concerned about a child’s clinical status, understanding these parental attributes may suggest strategies to improve adherence and health. Behavioral and psychological interventions to directly improve self-efficacy may lead to improved adherence [40, 41]. If a parent reports negative spiritual coping, addressing this through counseling may improve their ability to adhere to complex medication regimens. It is possible that the best interventions for these children and families are measures that attempt to directly increase adherence, including simplifying dosing frequency, addressing socioeconomic barriers, and improving health literacy [42, 43]. These findings may be reflective of what clinicians already experience in point of care and, as a result of this study, could serve as a more systematic means for clinicians to intervene. Negative spiritual coping correlated with decreased adherence to aerosolized medications and lower self-efficacy in parents. While causation is not established, this suggests that incorporating religious support for families experiencing these struggles may improve adherence and efficacy. While many multidisciplinary CF teams may not have a dedicated spiritual care specialist as part of core expertise, a clinical chaplain, for example, could be consulted. Further prospective, longitudinal studies that include trajectories of markers (e.g., longitudinal BMI) will be needed to understand if this association is causative and modifiable.

## Conclusion

Despite the study limitations, the results of this study indicate that parents of children with CF show personality traits that may be indicative of their adherence patterns and that these patterns can be learned through multivariate clustering methods. Clinicians may use analytic findings and clinical personas from this study as to understand how a given parent’s depression, anxiety, spirituality and complexity of routine, their child’s nutrition, and the distance they have to travel from home to their child’s CF care center can influence adherence. Care teams can use this framework to identify at-risk families for further interventions and personalized treatment plans of action. Use of these developments as decision support aids offer an opportunity to tailor and improve adherence to treatment regimens.

## Supplementary information

**Additional file 1.**

## Data Availability

Requests for further data not already available from this publication can be directed to Author DHG, who is the PI of the study. Email: DGrossoehme@akronchildrens.org

## Abbreviations

AC: Airway Clearance
Aero: Aerosolized Medication
BMI: Body Mass Index
CF: Cystic Fibrosis
DPD: Daily Phone Diary
MFA: Multiple Factor Analysis
PLSR: Partial least squares regression
