# Supplementary material for: Data driven decision making to characterize clinical personas of parents of children with cystic fibrosis: a mixed methods study
Source: BMC Pulm Med. 2020 Jun 18;20:174. doi: 10.1186/s12890-020-01202-x (PMC7301999; doi:10.1186/s12890-020-01202-x)
Supplement: Supplementary file 1 — Additional file 1. [file 12890_2020_1202_MOESM1_ESM.docx]

###### **Data Driven Decision Making to Characterize Clinical Personas of Parents of Children with Cystic Fibrosis: A Mixed Methods Study**

**Online Supplement**

Rhonda D. Szczesniak, Teresa Pestian, Leo L. Duan, Dan Li, Sophia Stamper, Brycen Ferrara, Elizabeth Kramer, John P. Clancy and Daniel Grossoehme

**Table S1: Checklist for reporting mixed-methods study***

| Manuscript section | Guideline | Inclusion in study article? |
| --- | --- | --- |
| Title | - Include broad terminology implying both quantitative and qualitative perspectives - Use term “mixed methods” | - Yes, in title - Yes, in title |
| Abstract | - Specify type of mixed methods design employed - Specify overall mixed methods approach to inquiry - Identify strategy used to integrate quantitative and qualitative data should be explicit - Results/Discussion reflect mixing of qualitative and quantitative data | - Yes, explanatory sequential - Yes, three phases (quantitative, qualitative and integration) - Yes, integration phase - Yes, mixed in conclusion section |
| Introduction | - Clearly identify gap in the literature; foreshadow use of mixed methods - Reflect how conceptual or theoretical perspective facilitates understanding of mixed methods - Support justification for why mixed methods approach is needed - Identify the three research questions (qualitative, quantitative, and mixed methods) - Provide clear basis for Methods, Results, and Discussion | - Yes, 1^st^-2^nd^ paragraphs - Yes, 2^nd^ paragraph - Yes, 3^rd^ paragraph - Yes, 4^th^ paragraph - Yes, 3^rd^-4^th^ paragraphs |
| Methods | - Use up-to-date references to demonstrate plan for data collection, analysis, and interpretation based on newest mixed methods typology - Identify and describe qualitative and quantitative measures, and demonstrate their contribution towards addressing the research questions - Analytic plan should identify mixed methods design and describe the qualitative analysis, and quantitative analytic methods, and type of data integration, where data integration will occur, and how integration is linked to the mixed methods design | - Yes, 1^st^ paragraph - Yes - Yes |
| Results | - Qualitative: include full thematic (or other) analysis - Quantitative: reflect a full analysis including hypothesis - Mixed methods: analysis shows results of data integration - Table, diagrams, and figures enhance presentation of all three types of data | - Yes, focus group and 1:1 interviews - Yes - Yes - Yes, Tables 1-3 (quantitative); Table 4 (qualitative and integration); Figure 1 (quantitative) |
| Discussion | - Include reflections on the results vis-a-vis the literature - Highlight new knowledge gained through investigation implications for future research, clinical practice, theory development, and policy (as applicable) - Highlight limitations - Explicate unique contributions of mixed methods to underscore potential importance | - Yes - Yes - Yes - Yes |
| References | - Include ample methodological articles | - Yes |

*directly adapted from Table II in (1).

**Table S2: Narrative format of clinical personas, scenarios and implications/actions***

| **Clinical Persona (Qualitative Phase)** | **Clinical Implications/Actions (Integration Phase)** |
| --- | --- |
| *Maria is a 26-year-old Hispanic woman who has been married for three years. They have a one-year old daughter, Beatrice, and they all live about 6 miles from the CF center. Maria typically wakes up around 7am and gives Beatrice her enzymes before they all eat breakfast together. After breakfast, Maria often naps or watches TV for 3-4 hours. Maria has minimal community support but gets a lot from her social media network. Around noon she prepares lunch and usually gives Beatrice her enzymes prior to eating. Beatrice’s first airway clearance is done around noon and then they often go outdoors for activities. Maria says “I don’t put her in a bubble, what’s the point? We don’t limit her in what we let her do because of CF.” Maria does not feel CF impacts her parenting. When they return home, they eat dinner as a family and afterwards Beatrice receives a treatment. She stays up until around midnight after Beatrice and Alonzo have gone to bed. Maria finds it difficult to get even half of Beatrice’s treatments completed. Maria hopes Beatrice will learn about CF and be responsible for her own treatments as she gets older. It is her hope that Beatrice is happy and grows up to have a family of her own. Although she does not see it, Maria has symptoms of depression, which have also contributed to her questioning her faith.* | *The immediate focus for this family would be on familial support. Medical intervention is not as important as mental health in this scenario. Because the family lives near the CF center, clinicians could offer monthly visits and coordinate with social workers and staff psychologists if applicable.* |
| *Liam is a 28-year-old Caucasian, stay-at-home, father of two-year-old Brian and 4-year-old Maddie whom both have CF. Liam and his wife treat every day like it’s their children’s last and try to fill it with fun activities. They both rely on social media for support and are not as active in the church. Liam wakes up both children to do their treatments. Liam and his wife’s anxiety about CF lead them to be diligent about getting medications and treatments on time. They eat breakfast as a family before taking both children to their CF appointments. After, they go to the park and play before returning home to do treatments and eat lunch. Having appointments for both children on the same day is necessary. They live far from the treatment center so having all appointments in one day makes it easier. Liam does chores around the house while the children play until it is time for treatments and dinner. When the children go to bed, Liam and his wife watch TV until they fall asleep. Liam wants his children to grow to be true to themselves and make good decisions and to live to an old age.* | *While this family has good adherence with CF, it seems they are anxious. It is concerning that that live everyday as if it is their last, this may sound like anxiety to a clinician. Clinicians could should partner with the family here to make care planning a team aspect. Partnering with the family in a medical setting can help normalize CF. Offering the family clinical research opportunities to enroll in can help alleviate some of the anxiety and give a sense of purpose. Making sure the family is properly connected with online groups is also important. With a myriad of information out there, it is imperative that the family is getting proper social media information and support. Connecting the family with a learning network that is monitored by the CF center can ensure that they are receiving proper support.* |
| *Anna is a 31-year old Caucasian woman who is happily married to Edward, with whom she has a 3-year old daughter, Francesca. They live about 15 miles away from the CF Center that follows Francesca. Anna wakes Francesca up and gets her ready for the day. She or Edward do Francesca’s first airway clearance of the day, Anna gives Francesca her enzymes, after which they eat breakfast as a family. Feeding Francesca isn’t much of a problem, and her BMI is always above the 50^th^ percentile. Edward leaves for work after breakfast. Anna has a Master’s degree in engineering and does consulting work from home while Francesca watches TV or naps. Sometimes Anna and Francesca run errands in the morning. Anna makes lunch, gives Francesca her enzymes, and they eat lunch together. Afterwards, Anna naps for two hours while Francesca naps as well. When Edward gets home, they cook dinner together, give Francesca her enzymes, eat dinner, and then do Francesca’s last airway clearance treatments. They watch TV together and do nightly prayers before bed. Anna says that getting Francesca’s treatments done is, “It’s a teamwork thing because sometimes I’m available to do treatments and sometimes I’m not.” In the evening, Anna says that the second treatment is “a routine. She gets to pick out a show she wants to watch and basically you just strap the vest on her. For the most part there is hardly any fuss.” While they have had to make some adjustments to their lives together because of Francesca’s CF, Anna says that, “CF fits in like anything else. I’m a germophobe, but I’m not OCD. That’s where my faith comes in. That’s a huge thing.” She and Edward are optimistic about Anna’s future with CF; Anna believes there’s going to be a cure within the next five to ten years. Anna’s major goals right now are just to try to get Francesca to feed herself and try to get her to do things more on her own. Anna’s long-term goals are for Francesca to get the best education that she can and be an outstanding adult and to enjoy her life.* | *This family is adherent and likely to incorporate changes if needed. This is the type of family that should continue to receive support, but there is not a strong need to intervene.* |
| *Barbara is a 35-year-old African American woman. She is married, but her marriage has some conflict especially around finances. They are parents to 7-year-old Henry and live about 30 miles from the CF center. Barbara wakes first in the morning then wakes up Henry. Sometimes she finds it difficult because she has her own health issues including depression. She makes breakfast but is not consistent about giving Henry his enzymes or doing his airway treatments afterwards. Henry is not always cooperative with his treatments, either. Barbara usually runs errands when she is not getting Henry to school on time. She sometimes remembers to give him his enzymes when they eat at home but deciding to eat out is often spur of the moment and she rarely remembers to bring his enzymes along. Henry’s BMI has always been in the 35^th^ percentile. They do a treatment at some time in the afternoon before Barbara naps. Henry likes to play video games and run around outside. If she didn’t do them before, Barbara will sometimes do Henry’s next treatment while cooking dinner. They eat dinner as a family before she prays and goes to bed alone. Barbara just, “…wants him to be happy. Hopefully he’ll find a really good job and a husband or wife, whichever way he goes, and to have kids and be a good person.”* | *It seems like this family is in chaos on how to work in day to day treatments. This family does not predict that they will make spur-of-the-moment decisions, so it is likely that is a reason they are forgetting to be adherent. It is important that the team focus on the most important medical outcome of the child as to not overwhelm this family. In this scenario, it is best to focus first on enzymes to increase BMI outcomes. Educating this family is important, but since they live further away from the CF center, it may be difficult for them to incorporate more appointments in their day to day schedule. Offering telehealth with a dietitian and social worker would provide this family with the support they need.* |
| *Charlie is a 30-year-old, married, Caucasian male. Together, he and his wife, have a 9-year-old daughter, Jacqueline who has CF and two daughters, aged 7 and 12, who do not have CF. In the mornings, Charlie wakes up and gets ready for work before waking Jacqueline to her treatments and take enzymes before breakfast and driving her to school. He says “CF is something we have to work around. The mornings are a little bit more difficult now, she wakes up, does her treatments, hurries and eats, then I take her to school.” Since Jacqueline is older now, it is easier because she is independent enough to do treatments herself. However, if she is feeling too sick or tired it can be more difficult to get them done. They have to plan their family activities around CF and make sure it is warm enough for her to be outside. Charlie notices that she tires easier than her sisters, so they have to watch out for Jacqueline more. Charlie goes to bible study once a week after work and he and his wife plan a date night once a week to make time for each other. They always make sure Jacqueline eats enough so they can keep her BMI above the 50^th^ percentile. They live near the CF center, but do not like when Jacqueline is hospitalized. He hopes Jacqueline understands the importance of eating and how much she needs to eat throughout her life. They say evening prayers every night as a family before bed.* | *This high functioning family does a good job of adhering to medication. Now that their daughter is becoming more independent, the team can focus on educating the child to do her own treatments. Fostering this independence can make for a smooth transition into adolescence and alleviate some stressors this family may face.* |
| *Danielle is a 43-year-old, married, Caucasian female. She and her husband have been happily married for 20 years. They live in a rural area about 55 miles from the CF center with their 11-year-old son, Luke. Danielle makes breakfast while her husband helps with Luke’s airway treatments. Because of his late diagnosis, it has been difficult to adjust to life with CF. Danielle says they work best with a routine, but even on the weekends when there is less of a routine, they still manage 85-90% of his daily treatments. After breakfast they get ready for the day and Danielle drops Luke off at school and goes to work. She picks him up from school and Luke does his treatments himself when they return home. His BMI is below the 50^th^ percentile, which concerns Danielle because she wants him to live a typical life like his friends. However, she is concerned because she wants him to be involved in some sort of activity, but a late CF diagnosis makes it difficult. Right now, she just wants Luke to graduate high school. When her husband returns at 7pm, they make sure Luke gets his enzymes and they eat dinner as a family. Luke does his nightly treatments before bed.* | *Normally, when people are nervous, clinicians can see high adherence. However, this family is still struggling with their son’s BMI. Using goal setting with providers and the child to incorporate him and the family onto the team could be helpful. This will also encourage independence with the child with appropriate oversight from the team.* |
| *Emma is a 37-year-old single mother to 13-year-old, Molly. Emma wakes up around 6am and wakes Molly to do her treatments while Emma gets ready for the day. They have a quick breakfast before Emma drops Molly off at school and goes to work. Sometimes, Emma has to leave work early to take Molly to the doctor. Living near the hospital has made this easier. Emma says ““Well, I just had to change my life a lot (since CF). The feasibility of keeping drugs on you seems to get harder and harder at school. I think we have control over it. She doesn’t really think that far ahead, she’s more day by day. CF is not a bad thing in our life, just part of our life. It’s a routine.” Emma and Molly do not get quite as many treatments done in a day as they would like, but they do 50-75% of them over the week. After school or appointments, Emma drops Molly off at cheerleading practice and waits until she is finished before grabbing fast food on the way home. Molly’s BMI is around the 4^th^ percentile. They have dinner while watching TV and say nightly prayers before bed. Emma hopes Molly gets into a routine and understands the importance of treatments.* | *It seems like this family has a good handle on their CF diagnosis. However, there is room for dietary improvements. Given their busy schedule, the team should focus on helping the family reschedule their routine and restructure their day to put more emphasis on medications.* |
| *Floyd is a 36-year-old Caucasian male who is happily married. He wakes up each morning and gets ready for work while his wife wakes up their son, 4-year-old Oliver, to do his treatments and she makes breakfast. After getting ready, Floyd eats breakfast with his family and then goes to work. When he returns, he plans with his son while his wife makes dinner. Floyd wants Oliver to grow up to be a sports announcer. Oliver’s BMI is around the 35^th^ percentile so he has difficulty doing things other kids his age do. Floyd feels like it is difficult to get treatments done, but understands that is just how it is, and just wants him to go to high school and college and live a typical life. After they play and eat dinner, Floyd helps his wife do Oliver’s last treatment. After Oliver and his wife go to bed, Floyd stays up until midnight doing work and watching TV. His main goal is to continue his job so he can have health insurance for his family.* | *Parents may come into the CF clinic and seem like they have high self-efficacy and are adherent. However, upon further probing it can be revealed that there are issues. Sometimes parents do not always think their lack of adherence is a problem because there is no visible evidence to support this. Offering a CT/MRI for evidence-based perception can show actual problems with non-adherence. Closer follow-up with this family is also recommended via monthly appointments and/or telehealth.* |

REFERECNCES

1. Wu YP, Deatrick JA, McQuaid EL, Thompson D. A Primer on Mixed Methods for Pediatric Researchers. J Pediatr Psychol. 2019;44(8):905-13.
